# Male factor testing in recurrent pregnancy loss cases: A narrative review

**DOI:** 10.18502/ijrm.v20i6.11440

**Published:** 2022-07-06

**Authors:** Soheila Pourmasumi, Parvin Sabeti, Nasrin Ghasemi

**Affiliations:** ^1^Social Determinants of Health Research Center, Rafsanjan University of Medical Sciences, Rafsanjan, Iran.; ^2^Clinical Research Development Unit (CRDU), Moradi Hospital, Rafsanjan University of Medical Sciences, Rafsanjan, Iran.; ^3^Department of Anatomy, Faculty of Medicine, Kurdistan University of Medical Sciences, Sanandaj, Iran.; ^4^Abortion Research Center, Yazd Reproductive Sciences Institute, Shahid Sadoughi University of Medical Sciences, Yazd, Iran.

**Keywords:** DNA fragmentation, Sperm, Y chromosome, Recurrent pregnancy loss.

## Abstract

Recurrent pregnancy loss is a distinct disorder defined as the loss of at least 2 pregnancies before the 20 ${}^{\text{th}}$ wk of gestation. With half of the genome of the embryo belonging to the father, the integrity of the sperm genome is crucial for a successful pregnancy. Semen analysis is recommended for men in such cases to evaluate sperm concentration, morphology, vitality and motility. However, other important sperm parameters such as sperm epigenetics, aneuploidy, Y chromosome microdeletion and chromatin integrity also correlate with successful pregnancy and delivery rate. This article examines the use of different sperm tests and their importance in male partners of women suffering from recurrent pregnancy loss.

## 1. Introduction

Men are accountable for 50% of the genomes of an embryo. However, they are usually not considered in clinical studies and the treatment management in recurrent pregnancy loss (RPL) cases (1). For many years, women have been held responsible for all forms of infertility and reproduction defects. Undoubtedly, women play an important role in reproduction and several complications may arise due to female infertility (2). Although many new clinical studies focus on the effect of male health on infertility and abortion (3). There are still not enough studies on the role of paternal factors in RPL (4). Moreover, very few studies have assessed and demonstrated the role of the male factor and its genetics in cases of recurrent abortion (5). The main diagnostic test to assess male fertility is a simple screening, including standard and routine semen analysis and some genetic testing; however, finding the best tests for diagnosing male infertility remains in debate (6). Paternal age is a known factor in cases of miscarriage, and older age in men can elevate the risk of spontaneous abortion (7). The hypothesis that men are fertile at any age while women's fertility is age-dependent is not totally correct (8, 9). Although, the relationship between advanced paternal age and the risk of spontaneous abortion is still unknown and controversial (10, 11). Some studies have already shown that the risk of abortion rises with the rise in men's age and decline in male fertility (12, 13). However, on the contrary, some studies have shown no increased rate of spontaneous abortion in cases with advanced paternal age (14, 15). Other risk factors involved in cases of male infertility are systematic and chronic disease (16), environmental and ecological toxins (17), obesity (18), and lifestyle parameters (19); however, in clinical trials, none of these factors have been thoroughly studied nor has the association between them and increased risk of spontaneous abortion in couples suffering RPL been evaluated.

Therefore, this article focuses on the different sperm tests that are currently available and their importance in determining male fertility in couples suffering RPL.

## 2. Materials and Methods

Scientific databases such as PubMed, Cochrane Library, and Scopus were searched for relevant articles published between 2009 and 2020 using the following keywords: pregnancy loss, recurrent pregnancy loss, recurrent abortion, miscarriage, male factor, paternal factor, sperm tests, and sperm DNA fragmentation.

The resultant articles were examined and suitable papers were selected for the current review. To determine the test results of men in RPL cases, the focus was placed on the laboratory sperm test outcomes of infertile and miscarriage cases. Of the selected studies, 17 studies examined the relationship between RPL and sperm DNA fragmentation (SDF), 7 evaluated the sperm chromatin by aniline blue (AB), toluidine blue (TB), acridine orange (AO), and chromomycin A3 staining (CMA3) staining, and 7 others evaluated the relationship between RPL and sperm aneuploidy. Additionally, 23 studies examined the relationship between RPL and sperm parameters, of which 19 were excluded due to duplication or differences in inclusion / exclusion criteria. The selected studies are listed in table I.

### Inclusion and exclusion criteria

Studies with cases who met the clear definition of RPL as 2 or more pregnancy losses were selected. In the selected studies, the maternal anatomical factors were measured by pelvic ultrasound, hysterosalpingogram, and sonohysterogram. The male factors such as alcohol consumption, drugs, smoking, and obesity were also investigated. On the other hand, studies that did not consider the precise definition of RPL and those with inaccurate and incorrect assessment of parental abnormalities were excluded.

### Laboratory tests

#### Semen analysis

Semen analysis is a basic fundamental laboratory test done to investigate the sperm parameters including concentration, motility, and morphology. This technique is usually used as a part of infertility assessment in men with poor sperm parameters and explains why some couples might fail to have a successful pregnancy (20). However, there is controversy regarding the efficacy of semen analysis in cases of miscarriage (21).

In some cases, the abnormal sperm morphology in men has been accepted as a possible etiology of an unsuccessful pregnancy, as fertilization with an abnormal sperm may not always result in a healthy embryo (22, 23). In addition, studies of RPL at infertility treatment clinics showed that abortion increased in couples with abnormal hypo-osmotic swelling scores (24, 25). It has been suggested that routine sperm analysis is insufficient for detecting sperm abnormality, and other functional individualities of sperms need more examination in RPL cases (26). Interestingly, some of the latest studies on sperm's role in RPL have reported no significant relationship between abnormal sperm parameters including count, morphology and motility, and the risk of RPL (27, 28).

Regardless, since several studies have shown that the standard sperm analysis alone is insufficient in RPL cases, many clinicians prefer to perform additional sperm tests. A few studies on sperm analysis and recurrent abortion are summarized in table I (29-31).

#### DNA fragmentation testing

There are several methods for detecting sperm DNA damage (20). However, based on some studies, the role of SDF in evaluating male fertility and in couples who suffer RPL is controversial (32). Many studies have shown that cases with RPL have a higher percentage of DNA fragmentation index (DFI) in the sperm, and this condition can affect sperm function, embryo formation, and development (1, 3, 4).

Some studies have shown that in cases with paternal advanced age, there was a higher percentage of DNA fragmentation (33, 34), varicocele, toxic exposure, alcohol consumption, and hormonal disorders (20). Moreover, some studies have shown that the level of DFI was higher in infertile men compared to fertile ones; however, documentations for men whose partner had a high risk of abortion were unpredictable (5, 35).

Some common SDF tests are the terminal deoxynucleotidyl transferase-mediated dUTP nick-end labeling assay (TUNEL), sperm chromatin dispersion test (SCD), and sperm chromatin structure assay (SCSA).

#### TUNEL assay

The TUNEL technique was presented for the first time by Gorczyza et al. in 1993. They found that this technique can detect the fraction in mammalian sperm DNA strands (1 or 2 strands) (36). In this technique, nucleotides are used to label the DNA strand breaks. Through this process, deoxyuridine triphosphate (DUTP) is modified by a terminal deoxynucleotidyl transferase called TDT, combined with biotin or digoxigenin at the 3 ' -OH end of broken string. Then the modified nucleotides are identified with a fluorescent antibody. Finally, an optical microscope, fluorescence, or flow cytometry systems can be used for assessing the fragmented DNA in the sperms (37). If sperms show bright fluorescent colors under the fluorescent microscope, this indicates that the sperms have fragmented DNA. Healthy and normal sperms without DNA fragmentation do not show fluorescent colors (20).

The TUNEL assay can detect DNA damage (single and double strand) and this test has a high sensitivity and reproducibility when using the flow cytometry method, but it requires fluorescence microscopy or flow cytometry, which are expensive, and it is a prolonged procedure with variable protocols (38). Many studies have used this assay to measure DNA damage in RPL patients (39-41).

Bareh et al. in a prospective, cohort study with TUNEL assay by flow cytometric analysis, indicated that the mean SDF was significantly higher in men with RPL (36.8 ± 5) than the controls (9.4 ± 2.7). They stated that despite the normal parameters in the semen analysis test, fragmentation of sperm DNA may be responsible in RPL cases with unknown cause (42). In another study, researchers investigated 112 men from RPL couples. They evaluated the SDF by TUNEL assay and indicated a correlation between increased SDF and impaired fertilization and pregnancies; however, they expressed that a high level of SDF still cannot reflect a predictive condition for elevation of the RPL risk (43). Other studies obtained similar results with the TUNEL assay (44, 45).

#### SCD

The SCD test is a simple and rapid assay. This assay is based on the halo test and is performed without any complex laboratory equipment. Spermatozoa enclosed in a thin inert matrix are treated with an acid solution which denatures the sperm DNA followed by a lysis buffer that removes the majority of nuclear proteins. As a result, it creates DNA loops spreading out into the inert matrix and forming halos of chromatin; however, sperms with fragmented DNA do not produce or show very small halos of DNA loop. Thus, this is a simple, inexpensive, and convenient technique. Also, a low number of sperm is needed. However, the assay only detects single-strand breaks with low contrasting images and an inter-observer subjectivity is needed for the interpretation of halos (38).

A study revealed the strongest association between TUNEL and miscarriage among all assays tested. The authors stated that the reason for this difference could be related to the lack of a denaturation step in the TUNEL assay (21). Another study showed that the SCD test in comparison with the TUNEL assay had high sensitivity for the assessment of DNA damage in unexplained infertile men (46). A systematic review demonstrated a significant strong relationship between defects in sperm chromatin quality, especially high levels of sperm DNA damage, and occurrence of RPL using both TUNEL and SCD assays. In addition, when they analyzed the subgroup and assessed the comparison between some sperm fragmentation tests, a similar association was seen with TUNEL and SCD by RPL risk (47). Studies measuring the DNA fragmentation of RPL patients using SCD assay are listed in table I (35, 40, 48-50).

#### SCSA

For preparing SCSA, the sperm sample is first mixed with low pH media or kept in a heated state to create a stressful situation for sperm to expose sperm DNA. After this, color is added to the sample which then attaches to the fragmented part of the DNA. If the DNA is intact and unbroken, color does not attach to it. The fragmented part of the DNA shines through a flow cytometer machine and the DFI is calculated. Clinical studies have shown that a DFI > 30% is related to fertility issues such as embryo development defects and pregnancy loss. The benefits of this test are that it can monitor a lot of sperm and it has a standard protocol, which reduces the disparity between different laboratories.

While standardization of SCSA has made this technique highly reliable, it needs expensive instruments (flow cytometer) and an expert laboratory operator (51, 52). In a case-control study, SCSA was used to characterize the level of DFI in 20 infertile men who were suffering from an unknown cause of RPL. The results of the DFI comparison showed that the mean DFI in the case group was significantly higher than in the control group (41). Likewise, several other studies have shown high levels of DNA fragmentation in RPL patients by SCSA assay (39, 53, 54).

#### Comet assay

A sensitive and rapid test that is used for detection of sperm DNA damage is single-cell gel electrophoresis (Comet assay). It is a suitable method for diagnosis between breached single- or double- DNA strands (ssDNA and dsDNA). In this technique, alkaline denaturing or neutral conditions are responsible for DNA damage detection (55). Researchers can evaluate the correlation between oxidative stress and enzymatic DNA damage, with high sensitivity, by using this technique (56). Few studies have been conducted with these assays in RPL patients. Yuan and colleagues in 2019 reported in RPL patients the level of DFI was significantly higher in comparison with normal donor control cases (57). In another study, Ribas-Maynou et al. described the ssDNA and dsDNA breaks by alkaline and neutral Comet assay. They suggested that ssDNA damage could be a predictor of fertilization but dsDNA damage is related to the risk of male factor-associated miscarriage which could be due to the lack of DNA repair by the oocyte (49).

#### Sperm chromatin/DNA evaluation

#### 2.2.7.1. AB staining

Histones are a combination of the amino acids lysine and arginine, which have a predominantly positive electric charge that binds to negatively charged DNA through electrostatic interactions. Lysine is alkaline and AB is an acidic dye that reacts with lysine. Using a light microscope, the sperm nucleus with a high level of histones can be seen in blue. During the last stage of spermatogenesis, the histone is replaced by protamine, so aniline staining shows immature sperm with a high level of histones. The results of this test, in a study conducted by Talebi et al., showed that the percentage of immature spermatozoa from an unexplained recurrent spontaneous abortions (RSA) group was higher than in the fertile men with no history of RSA using AB staining (3).

In another study, which aimed to investigate the effect of semen quality and lifestyle on recurrent abortion patients showed that the RPL group had significantly lower total sperm progressive motility, normal morphology, and viability, but a higher mean percentage of AB staining-positive sperm compared with the control group (58).

Nabi et al. in their study evaluated bacterial contaminations in the seminal fluid of male partners in unexplained RPL. They also used AB and TB staining to evaluate the sperm chromatin density in RPL and fertile groups. According to their findings, bacterial contamination was higher in RPL cases than in the fertile group. In addition, AB staining showed that the percentage of abnormal spermatozoa (AB+) in the RPL group was higher than in the control group (59). In a clinical trial performed on 60 RPL patients, the effect of vitamin E and zinc on sperm chromatin quality was investigated by AB, TB, and CMA3 staining. They found that the number of AB-positive sperm decreased significantly after antioxidant therapy (60).

#### 2.2.7.2. TB staining

TB (or tolonium chloride) is an acidophilic and metachromatic dye. This dye stains acidic tissue components such as phosphate groups and has a strong affinity for nucleic acids which by binding to phosphate groups of DNA can show the quality and quantity of sperm nucleus chromatin condensation and DNA fragmentation. With increasing abnormalities in sperm chromatin, more phosphate groups are exposed to staining and, as a result, a spectrum of blue color is obtained based on the degree of abnormality. With this test, sperm with normal chromatin turn light blue, those with some abnormal chromatin turn dark blue, those with abnormal chromatin turn violet, and those with highly abnormal chromatin turn purple (3, 60).

Results of a study indicated that sperm chromatin condensation and DNA integrity of male RSA patients were lower than the control group (3). Several other studies have examined the chromatin density of sperm in this group of patients (4, 59, 60).

#### 2.2.7.3. CMA3

During spermatogenesis, histones are replaced by protamines to form a dense structure and preserve the male genome health as the sperm passes through the female's genital system. However, normally about 15% of histones remain. But when the percentage of histones in sperm chromatin increases, or in other words, protamination decreases, it indicates that sperm chromatin is immature and can reduce sperm quality and fertilization power. CMA3 is an indirect method for assessing protamination. It is a fluorochrome that competes with protamine for binding to the minor groove of the DNA strand (61).

Several studies have used this test to evaluate sperm chromatin density in male partners of RPL couples, which are listed in table I (1, 3, 62).

#### 2.2.7.4. AO

AO is a simple fluorescence test used to distinguish sperm with normal chromatin from sperm with abnormal chromatin. Normal DNA in sperm is heterochromatic and very dense with many disulfide bonds that make its structure resistant to heat and chemical denaturation. Many authors have reported that the presence of > 50% of green fluorescent sperm in a sperm sample is the normal cut-off for this test in fertile specimens (63). Due to the reduced fluorescence property of AO after some time and the heterogeneity of staining in different areas of the slide, the use of this staining in very precise cases is low (62). However, a few studies have used this staining to differentiate normal dsDNA from denatured ssDNA in sperms of male partners of RPL couples (3, 62).

#### Epigenetics' role in sperm

Epigenetics is the assessment of noncoding changes in the content of DNA that do not alter the basic DNA sequence but play a regulatory role. Some of these changes are methylation, histone modification, and micro RNAs (64). Novel tests are examining the role of epigenetics and sperm function in infertility, and more studies must be done to assess the relationship between epigenetics and an RPL risk.

Poorang et al. in a case-control study investigated sperm parameters and methylenetetrahydrofolate reductase (MTHFR) epigenotypes of sperms in RPL cases. Although they found no statistically significant difference in the sperm methylated MTHFR epigenotype frequency and prevalence in RPL vs. non-RPL males, these was more frequent in men with abnormal sperm parameters. They also reported that the mutated allele of C677T was statistically higher in prevalence among RPL males (65). Another study assessed the relationship between paternal miR-196a2C > T and miR-499aT > C polymorphisms and the likelihood of RPL by tetra-primer amplification refractory mutation system-polymerase chain reaction (PCR) and PCR-restriction fragment length polymorphism for miR-196a2C > T and miR-499aT > C polymorphisms, separately. They observed a strong relationship between the likelihood of RPL and miR-499a (66). Likewise in some studies, the researchers assessed the effect of genetic frequency of paternal M2 in couples with RPL and compared it with fertile German controls. They reported a significant relationship between the genetic frequency of paternal M2 and RPL especially in the 10 ${}^{\text{th}}$ and 15 ${}^{\text{th}}$ wk of pregnancy (67, 68).

USP26 is a deubiquitinating enzyme which plays an essential role in the elimination of histones during spermatogenesis. In a recent study performed by Asadpor et al. it was confirmed that the mutation in 3 common haplotypes of the *USP26* gene in total frequency was significantly increased in the infertile cases and RPL group in comparison to the fertile controls. This may cause an increase in sperm DNA histone levels and subsequently increase sperm DNA damage (69).

Another study showed a higher level of protamine-1 and protamine-2 in spermatozoa of the RPL group compared to healthy sperm donors. They suggested that protamines may play an additional role in early embryogenesis (70).

#### Sperm aneuploidy testing

Chromosomal abnormalities in somatic cells can be discovered by a simple blood karyotype analysis. Errors during sperm meiosis lead to chromosomal abnormalities, which cannot be detected by a blood karyotype analysis (71). A common cytogenetic tool for sperm aneuploidy screening is fluorescent in situ hybridization (45). However, it is a time-consuming technique and laboratories cannot usually use it. Fluorescent in situ hybridization has some disadvantages for sperm aneuploidy; first, using this test, not all sperms can be assessed; second, in general, individually 13, 18, 21, X, and Y chromosomes can be checked. However, the demand for this technique is increasing before patients are referred to assisted reproductive techniques (72). A small number of studies have examined abnormal sperm aneuploidy as a possible reason for RPL. Findings of a study showed a higher level of chromosome 1 and 8 disomy and total aneuploidy in the RPL group (73). 2 other studies indicated an increased aneuploidy in men who experienced RPL compared with normozoospermic fertile men (74, 75). In another study designed by Neusser and colleagues, it was shown that meiotic errors that involve chromosome 16 can elevate disomy (higher than 60%) in RPL patients' sperm (76). Likewise, Esquerre-Lamare et al. found disomy in chromosome 18, hyperhaploidy, and total aneuploidy that were significantly elevated in the RPL group compared to the fertile controls (77).

#### Y chromosome microdeletion (YCM) analysis

YCM is a family of genetic disorders produced by missing genes or genes in the Y chromosome. For its analysis, blood leukocytes are used and confirmed with multiplex PCR (78). Some investigators have considered the prevalence of Y-chromosome microdeletions in RPL patients. Several studies have reported a significantly higher prevalence of YCM in RPL patients compared with the control group (79, 80); however, several studies noted no significant difference (81-83).

**Table 1:** Assessment of laboratory sperm tests in RPL cases


**Etiology/Reference**	**Conclusion**
**Semen analysis**	
**Kavitha ** ***et al.*** ** (31)**	Abnormal sperm parameters were found in 36.8% of RPL patients
**Zidi-Jrah** ***et al.*** ** (45)**	The sperm progressive motility was significantly lower and abnormal morphology was significantly higher in the RPL group vs. the fertile group
**Zhang** ***et al. *** **(50)**	There were no significant differences in sperm concentration, motility, and normal morphology between the RSA group and the controls
**Esquerre-Lamare ** ***et al.*** ** (77)**	Abnormal parameters (morphology and concentration) were found in 25% of the patients
**TUNEL assay**	
**Bareh ** ***et al.*** ** (42)**	The mean SDF was significantly higher in men with RPL compared with normozoospermic men
**Carlini ** ***et al.*** ** (43)**	A correlation between increased SDF and impaired fertilization and pregnancies was noted
**Brahem ** ***et al.*** ** (44)**	Men with a history of RPL had a higher incidence of DNA damage and poor motility than men from the control group
**Zidi-Jrah ** ***et al.*** ** (45)**	The percentages of SDF and nuclear chromatin decondensation were significantly higher in the RPL group than in fertile men
**Coughlan ** ***et al.*** ** (40)**	There were no statistical differences in the percentage of sperm with DNA damage between all groups
**Imam ** ***et al.*** ** (41)**	The mean DFI was significantly higher compared to the controls
**SCD**	
**Bellver ** ***et al.*** ** (35)**	The SDF was higher in the RSA group compared with the fertile sperm donors group
**Coughlan ** ***et al.*** ** (40)**	The SDF was not an important cause of RIF or RM
**Khadem ** ***et al.*** ** (48)**	The level of abnormal DNA fragmentation in the RSA group was significantly higher than in the control group
**Ribas-Maynou ** ***et al.*** ** (49)**	The SDF was significantly higher compared to in the controls (p < 0.01)
**Zhang ** ***et al.*** ** (50)**	There was no significant difference in sperm chromatin integrity between the RSA group and the controls
**SCSA**	
**Eisenberg ** ***et al.*** ** (53)**	Couples experiencing a pregnancy loss were more likely to have male partners with abnormal SDF
**Kumar ** ***et al.*** ** (39)**	Sperm from men with a history of idiopathic RPL had a higher percentage of DNA damage compared to the control group
**Venkatesh ** ***et al.*** ** (54) **	Male partners of RSA couples with normal sperm parameters had increased (p < 0.001) sperm DNA damage
**Comet assay**	
**Ribas-Maynou ** ***et al.*** ** (56)**	SsDNA damage could be a predictor of fertilization but dsDNA damage was related to the risk of male factor-associated miscarriage
**Yuan ** ***et al.*** ** (57)**	DFI was significantly higher in RSA patients compared with normal donor controls and there was only a weak partial correlation between DFI values and conventional sperm analysis parameters
**AB + TB + AO + CMA3 staining**	
**Talebi ** ***et al.*** ** (3)**	The percentage of immature spermatozoa from the unexplained RSA group was higher than in fertile men with no history of RSA using AB, TB, AO, and CMA3 staining
**Ruixue ** ***et al.*** ** (58)**	The RPL group had a higher mean percentage of AB staining-positive sperm compared with the control group
**Nabi ** ***et al.*** ** (59)**	The bacterial contamination was higher in the RPL group than in the fertile group. In addition, AB & TB staining showed that the percentage of abnormal spermatozoa (AB ${}^{+}$ ) & (TB ${}^{+}$ ) in the RPL group was higher than in the control group
**Nazari ** ***et al.*** ** (60)**	The effect of vitamin E and zinc on sperm chromatin quality was evaluated by AB, TB, and CMA3 staining. It was found that the number of AB, TB, and CMA3-positive sperm decreased significantly after antioxidant therapy
**Pourmasumi ** ***et al.*** ** (4)**	The effect of vitamin E and selenium on sperm chromatin quality in couples with RM was evaluated, and it was found that the number of TB, AB, CMA3-positive sperm decreased significantly after antioxidant therapy
**Kazerooni ** ***et al.*** ** (62)**	The sperm chromatin quality in couples with SRA was compared to the fertile group. The SRA group had a significantly higher percentage of CMA3 ${}^{+}$ and AB ${}^{+}$ sperm than the fertile men, but the AO results in the 2 groups did not differ significantly
**Talebi ** ***et al.*** ** (1)**	The RSA patients had a significantly higher percentage of spermatozoa with protamine deficiency compared to the fertile group by CMA3 test
**Epigenetics role in sperm**	
**Poorang ** ***et al.*** ** (65)**	No significant difference was observed in the frequency of methylated MTHFR epigenotype between RPL and non-RPL males. Also, the mutated allele of C677T showed statistically higher prevalence among RPL males
**Amin-Beidokhti ** ***et al.*** ** (66)**	A strong relationship between the chance of RPL occurrence and *miR-499a* was observed
**Rogenhofer ** ***et al.*** ** (67)**	Paternal M2 carriage seemed to confer an equal risk for RM as M2 carriage in RPL mothers
**Tuttelmann ** ***et al.*** ** (68)**	A higher prevalence of M2 haplotype in *ANXA5* was seen in male partners of German RPL patients
**Asadpor ** ***et al.*** ** (69)**	Mutation in 3 common haplotypes of the *USP26* gene in total frequency was increased significantly in the infertile cases and RPL group in comparison to the fertile controls
**Rogenhofer ** ***et al.*** ** (70)**	A higher level of protamine-1 and protamine-2 in the spermatozoa of the RPL group compared to the healthy sperm donors was noted
**Sperm aneuploidy testing**	
**Ramasamy ** ***et al.*** ** (75)**	Fluorescence in situ hybridization detected increased sperm aneuploidy in men with RPL
**Neusser ** ***et al.*** ** (76)**	Meiotic errors involving chromosome 16 contributed to increased sperm disomy in > 60% of RPL patients
**Esquerre-Lamare ** ***et al.*** ** (77)**	A significant increase in chromosome 18 disomy, hyperhaploidy, and total aneuploidy were observed in the unexplained RPL group compared with the fertile controls
**Agarwal ** ***et al.*** ** (80)**	The prevalence of the Y chromosome microdeletion in RPL patients was significantly higher than in fertile controls where no Y chromosome microdeletion was present
**Wettasinghe ** ***et al.*** ** (81)**	RPL patients had no microdeletions in the AZFa, AZFb, AZFc regions or partial deletions in the AZFc region
**Piña-Aguilar ** ***et al.*** ** (82)**	Results showed an absence of Y chromosome microdeletions in males of couples with RPL and controls with an acceptable statistical power
**Ghorbian ** ***et al.*** ** (83)**	None of the men in the case and control groups had any microdeletions in the AZFa, AZFb, and AZFc regions
RPL: Recurrent pregnancy loss, RSA: Recurrent spontaneous abortions, TUNEL: Terminal deoxynucleotidyl transferase dUTP nick end labeling, SDF: Sperm DNA fragmentation, DFI: DNA fragmentation index, SCD: Sperm chromatin dispersion test, RIF: Recurrent implantation failure, RM: Recurrent miscarriage, SCSA: Sperm chromatin structure assay, SsDNA: Single-stranded DNA, dsDNA: Double-stranded DNA, AB: Aniline blue, TB: Toluidine blue, AO: Acridine orange, CMA3: Chromomycin A3, MTHFR: Methylenetetrahydrofolate reductase, miR: MicroRNA, M2: Metaphase II, ANX: Annexin, USP: Ubiquitin specific peptidase, AZF: Azoospermia factors	

## 3. Conclusion

Considering the importance of sperm integrity in a successful pregnancy, semen analysis is recommended for men in couples suffering RPL to evaluate sperm concentration, morphology, vitality, and motility. Based on the literature reviewed in this paper, the evaluation of the integrity of the sperm DNA, sperm aneuploidy, and YCM may be useful in guiding the management of RPL cases. Since the relationship between sperm DFI and RPL is being investigated and has been presented in this article, it seems that SDF testing may be a valuable tool for RPL assessment in clinical centers before assisted reproductive techniques.

##  Conflict of Interest

The authors have no financial or nonfinancial conflicts of interest.
